# Wealth and Meanness

**Published:** 1881-05

**Authors:** 


### WEALTH AND MEANNESS.

I TELL you that, in nine cases out of ten, great acquired wealth lifts up in monumental testimony the meanness of its possessor. I knew two neighbors, old Californians, who had about equal fortunes. They were both old settlers, both rich, and both much respected. In that fearful year, 1852, when the dying and destitute immigrants literally crawled on hands and knees over the Sierra trying to reach the settlements, one of these men drove all his cattle up to the mountains, butchered them, and fed the starving. He had his Mexicans pack all the mules with flour, which at that time cost almost its weight in gold, and push on night and day over the mountains to meet the strangers there and feed them, so that they might have strength to reach his house, where they could have shelter and rest.

The other man, cold and cautious, saw his opportunity and embraced it. He sat at home, and sold all his wheat and mules and meat, and, with the vast opportunities for turning money to account in that new country, soon became almost a prince in fortune. But his generous neighbor died a beggar in Idaho, where he had gone to try to make another fortune. He literally had not money enough to buy a shroud, and as he died among strangers by the roadside he was buried without even so much as a pine-board coffin. I saw his grave there only last year. Some one had set up a rough granite stone at the head. And that is all. No name —not even a letter or a date. Nothing. But that boulder was fashioned by the hand of Almighty God, and in the little seams and dots and mossy scars that cover it He can read the rubric that chronicles the secret virtues of this lone dead man on the snowy mountains of Idaho.

The children of the "Prince" are in Paris. Upheld by his colossal wealth, their lives seem to embrace the universal world. He is my friend. He buys all my books, and reads every line I write. When he comes to this sketch he will understand it. And he ought to understand, too, that all the respect, admiration and love which the new land once gave these two men gathers around and is buried beneath that moss-grown granite stone, and that I know, even with all his show of splendor, that his heart is as cold and as empty as that dead man's hand.—*Joaquin Miller, in the Californian.*

---

A KANSAS weekly publishes "fourteen rules to be observed during a tornado." Only one is necessary: Be somewhere else.

